# Ultrasound Imaging of Thoracolumbar Fascia: A Systematic Review

**DOI:** 10.3390/medicina60071090

**Published:** 2024-07-03

**Authors:** Carmelo Pirri, Nina Pirri, Veronica Macchi, Andrea Porzionato, Raffaele De Caro, Carla Stecco

**Affiliations:** 1 Department of Neurosciences, Institute of Human Anatomy, University of Padua, 35121 Padova, Italy; veronica.macchi@unipd.it (V.M.); andrea.porzionato@unipd.it (A.P.); rdecaro@unipd.it (R.D.C.); carla.stecco@unipd.it (C.S.); 2 Department of Medicine—DIMED, School of Radiology, Radiology Institute, University of Padua, 35122 Padova, Italy; nina_92_@hotmail.it

**Keywords:** thoracolumbar fascia, low back pain, deep fascia, ultrasonography, aponeurosis, ultrasound, radiology

## Abstract

Over the past decade, there has been a notable increase in research focused on ultrasound imaging of thoracolumbar fascia (TLF). Nevertheless, published papers’ results about the application of US imaging in TLF examination are still sparse. *Background and Objevtives*: Hence, this systematic review was performed aiming to firstly investigate the use and the methodology of ultrasound imaging to assess pathologic and healthy TLF. Secondarily, we aim to assess intra- and inter-observer reproducibility of US imaging in TLF assessment. *Materials and Methods*: The search was done on PubMed and Web of Science database from inception to April 2024. Furthermore, the references of included papers were thoroughly checked to find eligible publications. The MeSH keywords used were: “Thoracolumbar fascia”, “Ultrasound Imaging”, “Ultrasound”, “Ultrasonography”, and “Ultrasound examination”. *Results*: Studies were aimed primarily at TLF diagnosis, treatment monitoring, or evaluating movement-related changes, underscoring the diverse clinical applications. The US parameters assessed included TLF thickness, echogenicity, stiffness, deformation, shear strain, and displacement, providing comprehensive insights into TLF features. *Conclusions*: Advanced US imaging holds promise as a reliable tool in musculoskeletal assessment, offering insights into TLF pathology/disfunction, treatment outcomes, and movement dynamics.

## 1. Introduction

Muscle–skeletal ultrasound (US) imaging is recognized as a safe, rapid, cost-effective, and widely accessible imaging technique, well-received by patients [1]. It enables through and dynamic assessments of the musculoskeletal system across multiple planes, offering detailed visualization of soft tissues with exceptional anatomical precision [2]. US use is increasingly embraced by physicians, with a continually expanding range of applications in physical and rehabilitation medicine, rheumatology, orthopedics, sport medicine, etc. Todorov et al. [3] highlighted the diagnostic role of US imaging in the evaluation of low back, reporting its function in the US examination of bony structure of the lumbar spine, intervertebral disc, sacroiliac joint, muscles, and soft tissues. 

The lumbosacral spine assumes a fundamental role in maintaining the body’s postural stability. However, the lumbar spine alone lacks the resilience to withstand the daily burdens it bears [4]. Providing support to the lumbar vertebrae atop the sacral base necessitates the involvement of an intricate network of myofascial aponeurotic structures that envelop the trunk [5,6,7]. At the core of this supportive framework along the posterior body wall lies the thoracolumbar fascia (TLF), a harmonious aponeurotic fascia that envelops the paraspinal muscles of the lower back and sacral region [8,9,10,11]. 

An increasing amount of evidence underscored the clinical importance of the TLF, a complex structure composed of layers of dense connective tissue located in the lumbar region of the trunk [5,6,7,8,9,10,11]. Research suggests that the fascia is integral in transmitting forces between the lower limbs and the trunk, as demonstrated in both ex vivo cadaver studies [8,9,10,11] and in vivo research during walking [12,13]. 

However, there is uncertainty regarding whether medical practitioners can reach a consensus on the diverse morphological features observed in US images of the TLF. The architecture of the TLF is complex, with layers of dense collagenous connective tissue interspersed with loose connective tissue, facilitating gliding movement and contributing to trunk mobility. Continuously connected with the aponeuroses of major trunk muscles essential for movement and vertebral control, the TLF is believed to undergo fibrosis, densification, and thickening in response to inflammatory processes or soft tissue injuries [13,14,15]. 

Nevertheless, published papers results about the application of US imaging in TLF examination are still sparse. Hence, this systematic review was performed aiming to firstly investigate the use and the methodology of ultrasound imaging to assess pathologic and healthy TLF. Secondarily, we aim to assess intra- and inter-observer reproducibility of US imaging in TLF assessment.

## 2. Materials and Methods

We conducted a systematic literature review following the guidelines outlined in the Preferred reporting Items for Systemic reviews and Meta-analyses (PRISMA) [16]. This systematic review protocol is registered in Open Science Framework registries with the registration https://doi.org/10.17605/OSF.IO/87RS5. The search for the literature was guided by the PICO (Problem/Patient; intervention/indicator, Comparison and Outcome) criteria detailed in Table 1.

The search was done on PubMed, Web of Science, and Scopus database from inception to April 2024. Furthermore, the references of included papers were thoroughly checked to find eligible publications. The MeSH keywords used were as follows: “Thoracolumbar fascia”, “Ultrasound Imaging”, “Ultrasound”, “Ultrasonography”, “Ultrasound examination”. The search strategy set for the topic was the following: (“Thoracolumbar Fascia”) OR (“Thoracolumbar Fascia” AND “Ultrasound Imaging”) OR (“Thoracolumbar Fascia” AND “Ultrasound”) OR (“Thoracolumbar Fascia” AND “Ultrasonography”) OR (“Thoracolumbar Fascia” AND “Ultrasound examination”). All relevant English-language publications were examined for potential inclusion, provided they demonstrated the involvement of US imaging in diagnosing or evaluating TLF. Criteria for exclusion were applied to avoid peripheral content, thus focusing on primary research efforts while still allowing for review papers, case reports, etc. The exclusion criteria were (1) papers focused on US-guided injection; (2) papers focused on surgery; (3) papers that did not discuss the use of US imaging for thoracolumbar fascia; (4) papers not published in English. 

**Figure 1:**
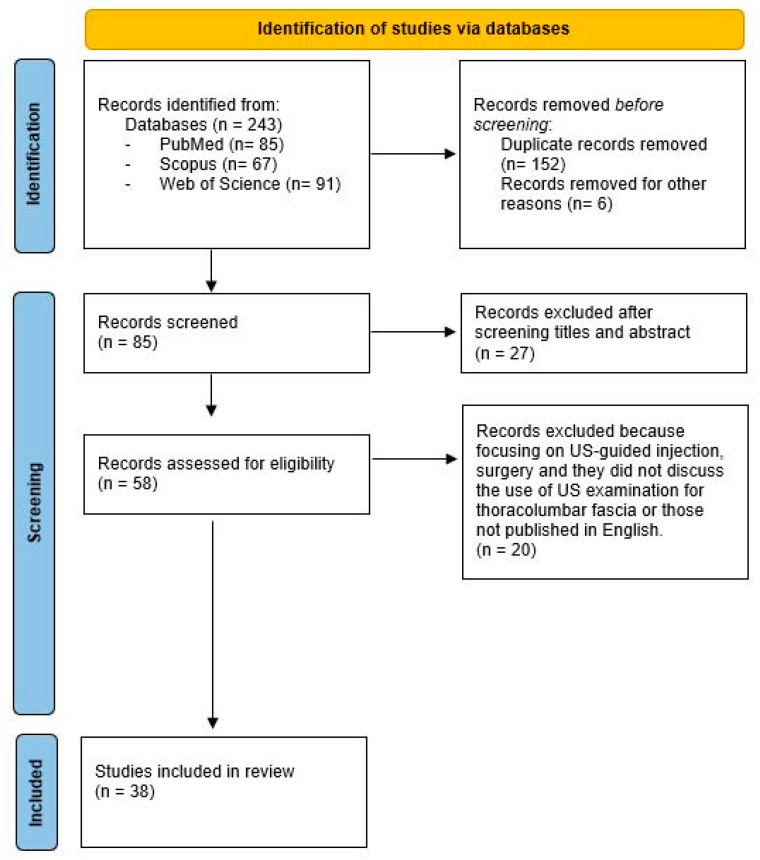
Flow chart of study selection.

We screened all studies by title and abstract, then the full text of eligible studies was checked for review, as well the references to identify any additional publications to be included. The literature search was carried out on by one reviewer (N.P.) and checked by one senior researcher (C.P.) with Ten years’ experience in ultrasound imaging of fasciae. Any discrepancy was resolved by agreement among the authors (Figure 1).

### 2.1. Data Extraction 

Data concerning these parameters were collected and analyzed:

- General characteristic of the paper: first author, year of publication, study design.
- Study population characteristics. Number of patients or healthy volunteers, age, gender, and TLF status (normal vs abnormal).
- Measurements methods: type of probe, type of US imaging, positions of patients or healthy volunteer.
- Reliability.
- Outcomes: evaluated parameters.

### 2.2. Risk of Bias 

Two researchers evaluated study quality and differences were solved after discussion. The papers were scrutinized for quality using the Risk of Bias Assessment tool for RCTs. This tool includes different domains of bias: patient selection, index test, reference standard, flow, and timing. Each domain was judged as “low risk”, “high risk” and “unclear”. The observational studies and case-control studies were assessed using Newcastle-Ottawa Scales (NOS), respectively, for observational studies and for case-control studies. The case-report studies were evaluated by JBI Critical Appraisal Checklist for Case Reports for case-report studies.

## 3. Results

Papers selected as regards the US imaging of TLF either in patients or healthy volunteers were analyzed. The agreement between the authors for including the articles was perfect (Cohen’s k = 0.90). The main characteristics of the studies (published between 2011 and 2024) are summarized in Table 2 [3,17,18,19,20,21,22,23,24,25,26,27,28,29,30,31,32,33,34,35,36,37,38,39,40,41,42,43,44,45,46,47,48,49,50,51,52,53]. Overall, 243 records were screened and 152 were removed because duplicates. Six were discarded. The text of the remaining 85 potentially eligible papers was checked, 47 of which were not in accordance with our inclusion criteria. Finally, we included 38 studies in the review. Figure 1 shows the flow diagram of our study selection.

### 3.1. General Characteristics of Studies 

According to their methodological design, most of papers included in the review were cross-sectional studies (28%; *n* = 11) [17,18,22,31,32,34,36,40,45,49,51]. The other studies were as follows: original articles (*n* = 6) [19,27,37,41,43,54], experimental laboratory studies (*n* = 4) [26,28,48,52], case-control studies (*n* = 3) [20,29,33], case reports (*n* = 3) [34,45,46]; randomized clinical trials (n = 3) [25,42,50], clinical trials (*n* = 2) [21,30], reviews (*n* = 3) [3,39,53], pilot studies (*n* = 1) [24], before and after experimental studies (*n* = 1) [23] and prospective studies (*n* = 1) [44] (Table 3). Most of the articles (85%) focused on ultrasound diagnosis, as the rest of articles (15%) assessed treatment benefits by ultrasound imaging. 

### 3.2. Type of Population 

Overall, the 34 papers included in the current review involved 740 healthy volunteers and 540 patients with pathological conditions; 497 male (44.33%) and 574 females (55.67%) had been studied, with an average age of 33.26 ± 11 years old. Most of participants were healthy volunteers and the remaining participants presented some clinical conditions, such as low back pain, gluteal pain, low back pain in scoliosis, etc. 

### 3.3. Assessed Fasciae and Other Musculoskeletal Structures

The papers included in this review addressed the deep fascial layers of TLF [3,17,18,19,20,21,22,23,25,27,28,29,30,33,35,37,38,41,42,45,46,47,48,50,51,52], the posterior layer of TLF [17,18,19,20,21,22,23,25,27,28,29,30,33,37,38,41,42,45,46,47,48,49,50,51,52], the superficial layer of the deep fascia of the back [22], the TLF and lumbar multifidus muscles [19,26], the TLF and erector spinae muscles [35], the TLF and diaphragm [24], the TLF and trasversus abdominis and internal oblique muscles [31,39], paravertebral muscles and perimuscular connective tissue [32], dorsal trunk and ventrolateral abdominal wall soft tissue [34,40], paraspinal muscular compartment [43], TLF and fascia lata and plantar fascia [44], TLF and various components of the lumbar spine [3,53], and TLF and semitendinosus (STF)/semimembranosus fascia (SMF) [18]. 

**Table 2:** Papers on ultrasound imaging of thoracolumbar fascia.

Authors and Year	Type of Paper	Number of Participants	Population	Sex	Age (Years Old)	Type of Anatomical Structure	Type of Probe (Frequency)	Type of US Imaging	Position	Parameters	Reliability	Aim
Yerli, S (2024) [17]	A cross-sectional study	60	Painful scoliosis, non-painful scoliosis and HV.	44 F and 16 M	16.3 ± 4y.	TLF	Linear probe	B-mode	Same protocol of [22]	Thickness	ICC: 0.84	To examine the thickening of the TLF was observed in subjects with scoliosis, whereby, in the presence of CLBP, it was further intensified.
Kellis E. (2023) [18]	Original article	14	HV	M	23.7 ± 7.31 y.	TLF, STF and SMF	(1) 4–15 MHz (2) 2–8 MHz	B-mode SWE	Measured at rest (passive condition) and during submaximal isometric knee flexion efforts (active condition) with the hip at neutral position and the knee flexed at 0°, 45°, and 90°.	- Thickness; - Stiffness	-	To examine the effect of passive and active knee flexion efforts on the stiffness of the TLF, STF and SMF.
Gumruk Aslan S. (2023) [19]	A cross-sectional study	50	- CLBP group (n = 30)—without LBP group (n = 20).	-	-	TLF LMM	-	B-mode	-	Thickness TLF and lumbar multifidus muscle	-	To quantitatively assess the thickness of TLF and LMM in younger-middle aged individuals, both those experiencing CLBP and those without LBP.
Brandl A. (2023) [20]	A case–control study	48	- Acute LBP (n = 16). - Two control groups: - UH, (n = 16); - FA (n = 16).	8 M and 8 F in each group	18–60 y.	TLF	Philips Lumify linear transducer 4–12 MHz	Cine B-mode	60-degree TL flexion controlled using a digital goniometer. Trunk extension over 8 s to the neutral position. Ultrasound TLFD measurement was performed in the starting and ending positions.	TLF deformation (TLFD) between the latissimus dorsi muscle junction and an artificial reference	ICC = 0.97	To investigate TLF deformation in athletes and non-athletes with and without acute low back pain.
Vining R. (2023) [21]	Clinical trial	40	Self-reporting LBP ≥ 1 year.	14 F 26 M	21–65 y. (mean 40 y.)	TLF	Terason 12L5 device, set at 10 MHz)	B-mode and CINE	Probe oriented parallel, 2–3 cm laterally to the L2–3 spinous process interspace at a point where target tissue layers were most visible. Prone-relaxed position on a table moving the lower extremities downward 15°, for five cycles at 0.5 Hz. To assess paraspinal muscle contraction effects, participants raised the head slightly from the table.	TLF shear strain	-	To assess TLF mobility and CLBP: Phase 1 of a pilot and feasibility study assessing repeated measures and the influence of paraspinal muscle contraction.
Pirri C. (2023) [22]	A cross-sectional study	92	- 46 CNLBP - 46 HV	47 F 45 M	CNLBP: 28.96 ± 10.54 y. HV: 27.09 ± 12.38 y.	TLF	Edge II, Sonosite, FUJIFILM, 6–15 MHz	B-mode	Relaxed prone position and the US transducer was placed parallel to the spine, approximately 2–3 cm lateral to the L3 spinous process	TLF thickness in the longitudinal and transverse axes	Intra-rater reliability: Long axis (CNLBP: ICC_(3,k)_: 0.91; HV ICC_(3,k)_: 0.92). Transverse axis:(CNLBP: ICC_(3,k)_: 0.88; HV: ICC3, k: 0.88).	To measure and compare by ultrasound imaging the thickness of the TLF at the bilateral L3 level of the lumbar spine in the longitudinal and transverse axes in chronic non-specific LBP and in healthy subjects, demonstrating an increase in non-specific LBP patients.
Devantery K. (2023) [23]	A before-and-after experimental study	49	LBP between 12th rib and gluteal fold for more than six months; - >3/10 NRS; - LBP > 50% of the time during the day.	25 M 24 F	>18 y.;	TLF ESM	Aixplorer Ultimate, SuperSonic Imagine, Aix-en-Provence, France; SL 10–2 MHz	B-mode; SWE	The probe was placed 2 cm lateral to L2–L3 interspinous space, on the right and left sides	Stiffness Thickness	-	To evaluate the immediate effect of a standardized versus a simulated MFT on the stiffness of the TLF and ESM using shear-wave elastography.
Perez M. A. (2023) [24]	A pilot study	54	NSLBP (n = 23) HV (n = 31)	NS-LBP: 10 M 13 F; HV: 12 M 19 F	18–60 y.	TLF Diaphragm	Vinno E35 (VINNO Technology, Suzhou, China) 7–18 MHz, with 38 mm footprint; Convex probe 1–5 MHz was used with 52 mm footprint.	B-mode (TLF) M-mode (diaphragm)	L4 vertebral level longitudinally over the anterior subcostal region in a supine position (bed slope of 45°)	Thickness of the TLF Diaphragmatic excursion	*-*	To perform a comparison based on the measurement of ultrasonographic parameters of the diaphragm, the lumbar multifidus muscles, and the TLF in subjects with and without NS-LBP.
Yang C. (2023) [25]	Randomized Controlled Trial	66	- PT group received 15 min of BPM - control group	M	22 ± 4.1 y.	TLF	A Mindray M7 scanner with a 4 cm, 10 MHz linear probe	B-mode	The probe centred at 2 cm lateral to the middle of the L2–L3 interspinous ligament	TLF thickness and echo intensity, perceived stiffness, lumbar flexibility, and skin temperature	Intra-observer: ICC = 0.95; inter-observer: ICC = 0.91.	To investigate the effects of PT on TLF morphology and other related outcomes.
Larivière C. (2023) [26]	Experimental laboratory study	70	CLBP	-	-	LM	-	B-mode	-	LuM echogenicity at three vertebral levels (L3/L4, L4/L5 and L5/S1); TLF posterior layer thickness PMCT thickness of the fasciae between STT and EO, between EO and IO, between IO and TrA, and between TrA and IA.	-	To explore whether these RUSI parameters (LuM echogenicity and fascia thicknesses), here after called dependent variables (DV) were linked to independent variables (IV) such as (1) other RUSI parameters (trunk muscle thickness and activation) and (2) physical and psychological measures. RUSI measures, as well as a clinical examination comprising physical tests and psychological questionnaires.
Tamartash H. (2023) [27]	A cross-sectional study	131	68 LBP 63 HV	LBP:33 M 35 F HV: 32 M 31F	40.2 ± 5.3 y. 41.7 ± 4.9 y.	thoracolumbar fascia (TLF)	SONON Ultrasound Imaging System with a 5–14 MHz linear probe.	B-mode and SWE	Prone position and placed their upper limbs in a relaxed position next to the body. The probe left side of L2–L3 vertebrae. These images were recorded “with stress” and “without stress” to achieve the elastic modulus of the TLF.	Strain of TLF	-	To evaluate the changes in the elastic behavior of LF in patients with CNLBP based on ultrasound imaging
Bartsch K. (2023) [28]	Experimental study	1	Multi-layered phantom model	-	-	TLF	Philips Lumify with L12–L4 linear transducer	B-mode	In two states: with stress and without stress. For the stress state scenario, compressive stress is imposed by the ultrasound transducer.	Tissue stiffness and Stress	ICC _(2,2)_ = 0.75–0.98	To compare different stiffness measurement tools reliability on a multi-layered phantom tissue model (MPTM).
Brandl A. (2022) [29]	Case-control study	10 with LBP; 10 HV	Acute LBP were matched to HV	LBP: 4 M 6 F HV: 4 M 6 F	43.6 ± 15.9 (LBP group) 39.0 ± 15.0 (control group)	TLF	Mindray DP2200, linear transducer 75L38HB, 5–10 MHz, sampling rate 7.5 MHz	B -mode and dynamic US	The transducer was then moved laterally along the line from the L1 spinous process in the sagittal section until the junction of the LD muscle with the TLF was visible.	Deformation of TLF defined by the distance between the intersection of the artificial reference and the underside of the PLF and the muscle–fascia junction of the LD and the TLF.	-	To reveal time-dependent relationships between biomechanical and neuromotor factors.
Vining R. (2022) [30]	clinical trial	20	CLBP following spinal manipulation and over an 8-week course of multimodal chiropractic care.	11 F 9 M	21–65 y. (mean 40 y.)	TLF	Terason T3000 ultrasound system with a transducer set at 10 MHz and programmed to record a cine-loop for 20 s in B-mode at a 25 Hz frame rate.	B-mode and cine	Ultrasound imaging occurred 2–3 cm lateral to L2–3 while participants relaxed prone on an automated table moving the lower extremities downward 15 degrees, for five cycles at 0.5 Hz.	TLF shear strain and TLF mobility	-	To assess TLF shear strain in persons with chronic low back pain following spinal manipulation and over an 8-week course of multimodal chiropractic care.
Turan Z. (2022) [31]	Cross-sectional study	30	HV	15 M; 15 F	28.8 ± 8.1 y.	Transversus abdominis and internal oblique muscles	Esaote MyLab Class C ultrasound device equipped with 55 mm convex transducer (CA 541, B-mode, frequency 5 MHz).	B-mode	Evaluated using ultrasound during four positions (rest, abdominal hollowing, bridge, and bridge with arm extension).	Thickness of transversus abdominis and internal oblique muscles	-	To evaluate the changes in the ultrasonographic thickness of transversus abdominis and internal oblique muscles during bridge with arm extension compared to bridge and abdominal hollowing.
Ushida K. (2022) [32]		17	CLBP	-	-	Paravertebral muscles and perimuscular connective tissues	-	B-mode	Measurements located lateral to the midpoint between L2-3 and L4-5 spines.	Thickness and echogenicity of the paravertebral muscles and PMCT.	-	To investigate the relationship between paravertebral muscles and PMCT of the TLF region and the four types of pain in patients suffering from CLBP.
Venkatesan P. (2022) [33]	Case-control study	144; Experimental group: yoga; Control Group: exercise based on DNS	Lumbar muscle in CLBP for longer than 3 months	18–45 y.	-	TLF	-	B-mode	TLF thickness	-	-	To compare the effects of yoga and dynamic neuromuscular stabilization exercise on CSA, fat infiltration of LMM with magnetic resonance imaging, and TLF thickness using musculoskeletal ultrasound imaging in CLBP.
Larivière C. (2021) [34]	Cross sectional study	64	34 LBP 30 HV	15 M 15 F	18–65 y.	Dorsal trunk and ventrolateral abdominal wall soft tissues	A 5–2 MHz curvilinear array transducer for lumbar spine, while a 12–5 MHz 50-mm linear array transducer for PMCT of the abdominal wall.	B-mode	USI measures were collected at rest on an exam table, in supine and prone positions), standardized task to assess muscle activation.	Muscle thickness, PMCT thickness; STTABD over the lateral abdominal wall.	ICC_(3,1)_ ¼ 0.92 and 0.96 for left and right sides, respectively.	To test the medium-term (8 weeks) test-retest reliability of the corresponding RUSI measures.
Pirri C. (2021) [35]	Case reports	1	Sedentary work at computer	F	35 years	TLF	Linear 4–16 MHz and convex 1–7 MHz	B-mode	-	-	-	To identify the reason of LBP.
Larivière C. (2021) [36]	Cross sectional study	64	30 HV and 34 LBP	HV: 15 M 15 F LBP: 15 M 18 F	18–65 y.	Dorsal trunk and ventrolateral abdominal wall soft tissues	5–2 MHz curvilinear array transducer (6.5 cm footprint), while the PMCT of abdominal wall using a 12–5 MHz 50-mm linear array transducer.	B-mode	The lumbar spine structures were imaged in the parasagittal plane	(1) Lumbar multifidus (LM) echogenicity at three vertebral levels (L3/L4, L4/L5 and L5/S1); (2) PLF thickness of the thoracolumbar fascia; (3) Thickness of the fasciae surrounding EO, IO and TrA.	-	To identify the main potential determinants of US measures of LM muscle fatty infiltrations, TLF thickness and thicknesses of PMCT surrounding the abdominal wall muscles.
Chen B. (2021) [37]	Cohort study	20	HV	M	18.4 ± 0.7 y.	TLF	Ultrasound transducer	SWE	Seven postures. (1) Rest, (2) sitting, (3) sitting-forward 30°, (4) sitting forward 60°, (5) standing, (6) standing-forward 30°, and (7) standing-forward 60°.	Stiffness	-	To use SWE to study the relationship between shear modulus and different body postures of TLF. Acquire physiologically meaningful information from the stiffness-posture graph to better quantify passive flexion responses.
Wakker J. (2021) [38]	Prospective clinical trial	267	HV	166 F; 101 M	36.1 ± 15.5 y.	TLF	Siemens Acuson S3000 TM 4.-9 MHz linear transducer.	B-mode a colour-coded elastogram, was positioned in the TLF	Lying prone with the arms adjacent to the body.	Stiffness	Intra-rater reliability ICC was between 0.857 and 0.979. The ICC for the inter-rater reliability was 0.931.	Determining the normal values for acoustic radiation force impulse (ARFI) SWE of TLF and define possible factors of influence.
Cheung W.K. (2020) [39]	Review	-	LBP and HV	-	-	TrA; MF + TLF	5- to 7-MHz linear, 5-MHz curved and 2 to 5 MHz curvilinear arrays	B-mode DOPPLER SWE	TrA thickness at the end of expiration; TLF: linear probe longitudinally 2 cm lateral to the midline at the level of the L2–3 interspace. MF: linear probe at 4 cm lateral to L3 over the longissimus muscle group.	Thickness, Doppler and Stiffness.	The ultrasound measurements had moderate and good between-day inter-rater reliability.	To highlight the current understanding of how medical ultrasound has been used for diagnosis and study of low back pain and discusses potential new applications.
Larivière C. (2020) [40]	Cross sectional study	64	30 HV 34 LBP	HV: 15 M 15 F/LBP: 15 M 18 F	18–65 y.	Dorsal trunk and ventrolateral abdominal wall soft tissues	A 5–2 MHz curvilinear array transducer with 6.5 cm footprint for lumbar spine; a 12–5 MHz 50-mm linear array transducer for PMCT of the abdominal wall.	B-mode	Images were collected on an exam table, in supine (ventrolateral abdominal wall) and prone (dorsal soft tissues), just before and during an isometric standardized task to induce muscle activation.	Thickness	-	To compare three quantitative measures of these tissues, using US imaging.
Chen B. (2020) [41]		20	Healthy	M	18.4 ± 0.7 y.	TLF	Aixplorer ultrasound device with a 40 mm linear array 10–2 MHz.	B-mode SWE	At the L3–L4. Horizontally 2 cm from the right side of the L2–3 and the L3–4 midline. All subjects performed in postures: sitting, sitting-forward 60°	Elasticity	Intra-operator (ICC = 0.860–0.938) and inter-operator (ICC = 0.904–0.944)	To examine the intra and inter-operator reliability of SWE device in quantifying the shear modulus of TLF and the device’s abilities to examine the shear modulus of the TLF during upper body forward.
Ünal M. (2020) [42]	Randomized Controlled Trial	40	CLBP	-	25–65 y.	TLF	Siemens Acuson X 700 and a Linear 10.7 MHz probe	B-mode	On the right and left sides of the dorsum with the patient in the prone position	Morphological structure of TLF.	-	The aim of this study was to comparatively investigate the effects of MIT against PNE on pain and function in patients with CLBP.
Blain M. (2019) [43]		15	HV, right-handed	6 F; 9 M	24 ± 4 y.	Paraspinal muscular compartment (PMC)	-	B-mode SWE	The transducer was oriented longitudinally centred at L3–L4 level, at 2 cm from the midline bilaterally. Performed in 5 postures including various trunk and arm positions.	Stiffness	ICC showed good to excellent intra-rater reliability.	The aims of this study were (1) to test the reliability of SWE in MFM and ESM in prone and sited position; (2) to investigate the role of the tensioning of the pTLF, via stretching of LD, on LPM stiffness.
Vita M. (2019) [44]	Prospective study	29	17 users, and 12 nonusers of hormonal contraceptives	F	18–29 y. (mean, 22.5 years)	TLF, FL and PF	SuperSonicAixplorer ultrasound machine with a linear array transducer SL 15–4 MHz.	B-mode SWE	Relaxed prone position with hands placed beside their thighs. The examined side respected the dorsal myofascial line.	Thickness Stiffness	-	To examine the influence of hormonal changes during the menstrual cycle on deep fasciae.
De Coninck K. (2018) [45]	Cross-sectional study	30	21 medical doctors, 7 physiotherapists and 2 radiologists.		13.03 ± 9.6 y. of experience	TLF	18 MHz linear array transducer (Esaote LA435)	B-mode	Intervertebral level 2–3, as fascial planes are the most parallel to the skin at this level.	Architectural disorganisation of TLF	-	To determine the inter-rater reliability for the rating of morphological characteristics of thoracolumbar fascia in ultrasound images, on Likert-type scale, by a range of clinicians.
Fullerton B. D (2018) [46]	Cases report	2	(1) >10 y. LBP (2) 5-m. right LBP and gluteal pain radiating to calf.	(1) M (2) F	(1) 48 y. (2) 52 y.	TLF	Linear-array high frequency transducer	B-mode	Prone position	Thickness of the TLF	-	To identify the alteration in TLF.
Panagos A. (2018) [47]	Case report	1	Chronic LBP	M	65 y.	TLF	-	B-mode	Right paraspinal muscles at the L5-S1 vertebral body level.	Thickness.	-	To identify the reason of LBP.
Todorov PT. (2018) [3]	Review	-	Patients with low back pain (LBP)	-	-	Lumbar and pelvic ligaments, muscles and entheses, TLF and the sacroiliac joints	10 MHz linear transducer	B-mode	Linear transducer in the longitudinal plane on a point 2 cm lateral to the midpoint at L2–L3 level.	Thickness and echogenicity	-	To review the literature on the diagnostic value of US in different conditions that could cause LBP.
Langevin H.M (2018) [48]	Animal study	20	Swine	-	4–6 wks	TLF	Terason 3000 scanner with a 4.0-mm, 10-MHz linear array transducer	B-mode + cine	L3–4 level in transverse axis, and the edge of the probe aligned with the lateral border of the vertebral body. Cine recording acquired during passive flexion of the trunk.	Thickness, Shear Strain and tissue displacement within the connective tissue layers of the TLF.	-	To determine whether the abnormalities in fascia mobility caused by an unilateral TLF injury and movement restriction can be reversed by removing the movement restriction, with or without the implementation of daily stretching for one month.
Wong KK. (2017) [49]	Cross-sectional study.	10	Healthy	M	22.8 ± 2.0 y.	PLF	Terason t3000 system, with a 5–12 MHz and 38 mm linear-array transducer	B-mode	Prone position with the shoulder internally rotated, palm up, elbow extended, and head in neutral position performed a press-down to MVC in the prone position.	Deformation of the PLF.	Moderate to good reliability of all parameters ICC _(3,3_) ranging from 0.95 to 0.98.	To quantify the immediate effects of MR on fascial properties of the PLF in healthy men.
Griefahn A. (2017) [50]	Randomized and controlled trial	38	Healthy athletic active	25 M 13 F	Mean 23.34 y.	TLF	A MyLab One Esaote, ultrasound machine with a 13–6 MHz linear probe	Cine B-mode	Sit position on the treatment table with their feet having contact to the ground and exercise the TL flexion, probe located 2 cm lateral and to the right of the spine, at L2–L3.	To calculate how displaceable the various layers of the TLF are.	ICC ranging from 0.79 to 0.9 and 0.76 to 0.79	To determine whether there is a significant difference in the mobility of the TLF among three treatment groups.
Tu S.J. (2016) [51]	Observational Study	12	HV	8 M 4 F	22.9 ± 3.59 y.	TL tissue	Voluson i, GE with a frequency 4–12 MHz linear probe.	B-mode + cine	Transducer 3 cm lateral to the middle of the L2 and L3 spinous processes; patients perform speed-guided lumbar flexion-extension tasks in two states (without taping and with KT).	Lumbar tissue movements	-	To assess the impact of KT on the movements of the TLF tissue.
Bishop J H (2016) [52]	Experimental design	20	Castrated male domestic swine	-	4–6 weeks old	TLF	Terason 3000 scanner with a 4.0 mm, 10 MHz linear array transducer	B-mode + ultrasound cine-recordings	Bilaterally at L2–3, L3–4 and L4–5 levels with the ultrasound probe oriented transversely, and the edge of the probe aligned with the lateral border of the vertebral body	Tissue displacement: during passive flexion of the trunk + tissue thickness	-	They used a porcine model to test the hypothesis that similar ultrasound findings can be produced experimentally in a porcine model by combining a local injury of fascia with movement restriction using a “hobble” device linking one foot to a chest harness for 8 weeks.
Darrieutort-Laffite C- (2014) [53]	Review	-	-	-	-	TLF and the various components of the lumbar spine	Transducer frequencies ranged from 2 to 9 MHz; linear 3–11 MHz probe, with the trapezoid mode when needed to expand the field of view.	B-mode	The transducer is placed on the midline, along the spinous processes, in the longitudinal direction. Seated or bent forward or in the prone position with a cushion under the abdomen.	-	-	To discuss a systematic approach to the ultrasonographic assessment of the lumbar spine.
Langevin H. M (2011) [54]	Original article	121	50 no—LBP 71 LBP ≥ 12 months duration	24 M 26 F 38 M 33 F	44.6 ± 1.8 41.8 ± 2.3	TLF	Terason 3000 ultrasound machine; 10 MHz (12L5) linear array transducer	B-mode + cine	Bilaterally of the back during passive trunk flexion using a motorized articulated table with the hinge point of the table at L4–5 and probe located longitudinally 2 cm lateral to the midline at the level of the L2–3 interspace	Displacement within TLF; PMCT thickness and echogenicity	-	To quantify shear plane motion within the TLF using ultrasound elasticity imaging in human subjects with and without CLBP.

y.: years; F: female; M:male; m. = months. N.s.: non specified. CLBP: chronic low back pain. LBP: low back pain. CNLBP: chronic non-specific low back pain. HV: healthy volunteers. SWE: shear-wave elastography. STF: semitendinosus fascia. SMF: semimembranosus fascia. LMM: lumbar multifidus muscle. UH: untrained healthy individuals. FA: field athletes. TLFD: thoracolumbar fascia displacement. NRS: numerical rating scale. ESM: erector spinae muscles. LF: lumbar fascia. LuM: Lumbar Multifidus. PMCT: Perimuscular connective tissues. STT: subcutaneous tissue thickness. EO: external oblique muscle. IO: internal oblique muscle. TrA: transversus abdominis muscle. IA: intra-abdominal content. PMC: Paraspinal muscular compartment. FL: fascia lata. PL: plantar fascia. PLF: posterior thoracolumbar fascia. LD: latissimus dorsi. MR: myofascial release. MVC: maximal voluntary contraction. KT: kinesiotaping. STTABD: subcutaneous tissue thickness. MIT: myofascial induction therapy. PNE: pain neuroscience education. BPM: back percussion massage. Wks: weeks.

**Table 3:** General characteristics of the 37 papers included in our analysis.

Type of Studies	N
Cross-sectional study	11
Original article	6
Experimental laboratory study	4
Case-control study	3
Case report	3
Randomized clinical trial	3
Clinical trial	2
Review	3
Pilot study	1
Before and after experimental study	1
Prospective study	1

### 3.4. US Equipment Charcteristics and Type of Probe

Across the studies included, multiple US devices were used, each equipped with linear or curvilinear array transducers. These devices featured distinct central frequencies and operated in various modes including B-mode [3,17,18,19,20,21,22,23,24,25,26,27,28,29,30,31,32,33,34,35,36,38,39,40,41,42,43,44,45,46,47,48,49,50,51,52,53,54], M-mode [24], or B-mode with elastography in particular shear-wave elastography [18,23,27,37,38,39,41,43,44]. The data indicated that B-mode was the predominant mode used across the majority of studies, followed by B-mode with elastography. Moreover, the TLF was evaluated with linear array transducers [3,17,18,20,21,22,23,24,25,26,27,28,29,30,34,35,36,38,39,40,41,43,44,45,47,48,49,50,51,52,53,54] and curvilinear array transducers [24,26,31,34,35,36,39,40,53]. Most articles provided the frequency ranges of the ultrasound transducers, which typically in the average varied from 4 MHz to 15 MHz for the linear probe and from 2 MHz to 7 MHz for the curvilinear probe. However, specific acquisition frequencies were rarely documented. Finally, some studies dynamically evaluated the TLF recording cine-loop up to 20 s at different frequency of frame rate [29,30,48,50,51,52,54]. 

### 3.5. Positioning of Patient and Protocol 

Several procedures were used to evaluate the TLF and TLF in relation with other musculoskeletal structures. In most studies, it was assessed with the subject in prone position [17,18,19,20,21,23,27,30,32,38,39,41,42,43,44,45,46,49,51,52,53,54]; other studies in association with different tasking. Kellis et al. [18] measured at rest (passive condition) and during submaximal isometric knee flexion efforts (active condition) with the hip at neutral position and the knee flexed at 0°, 45°, and 90°. Brandl et al. [19] performed a 60-degree thoracolumbar flexion which was controlled using a digital goniometer. Subsequently, the subjects extended the trunk over 8 s to the neutral position. Ultrasound measurement of TLF was performed in the starting and ending positions as in deadlift. Dynamic ultrasound measurements of the TLF displacement between the latissimus dorsi muscle junction and an artificial reference using a reflective tape were performed in the starting and ending positions [20]. Vining et al. [21] oriented the probe parallel, and 2–3 cm lateral, to the L2–3 spinous process interspace at a point where target fascial tissue layers were most visible. The participants lying prone and relaxed on a table moving the lower extremities downward 15°, for five cycles at 0.5 Hz. Pirri et al. [22] showed a protocol in which the patient was relaxed in the prone position and the US transducer was placed parallel to the spine, approximately 2–3 cm lateral to the L3 spinous process. Also, Devantery reported a protocol in which the probe was placed 2 cm lateral to L2–L3 interspinous space, bilaterally [23]. Perez et al. [24] reported an ultrasound evaluation at L4 vertebral level longitudinally over the anterior subcostal region in a supine position (bed slope of 45°). Chen et al. [40], Blain et al. [43], and Langevin et al. [48] also used the L3–L4 lumbar vertebra levels. Bishop et al. [52] and Langevin et al. [54] also assessed TLF at L4–L5 lumbar level while Panagos et al. [47] at L5-S1 vertebral level. The best point to evaluate the TLF was L2–L3 [21,22,23,27,30,32,38,39,41,45,49,51,52,53,54]. Chen et al. [37] evaluated TLF in seven postures (rest, sitting, sitting-forward 30°, sitting-forward 60°, standing, standing-forward 30°, and standing-forward 90°.) 

### 3.6. Parameters Evaluated with the Measurements

A multiplicity of parameters was assessed, including thickness [3,17,18,19,22,23,24,25,31,32,33,34,39,40,44,46,48], echogenicity [3,25,26,32,36], architectural disorganization [45], and stiffness [18,23,28,37,38,41,43,44]. The TLF deformation [20,29,49], TLF shear strain [21,27,30,48], and TLF displacement were evaluated in a dynamic way [50,52,54]. 

### 3.7. Reliability 

Thirteen of the fifty-five papers in this review analyzed the reliability of the US TLF measures [17,20,22,25,28,34,38,41,43,49,50]. Thirteen studies assessed the intra-rater reliability [17,20,22,25,28,34,38,41,43,49,50], while only three papers reported the evaluation of inter-reliability [25,38,41]. All the studies that evaluated the intra-rater reliability reported overall good to excellent reliability (ICC = 0.75–0.98). In addition, the studies about inter-rater reliability reported an excellent reliability (ICC = 0.93). 

### 3.8. Aims of Studies 

A multiplicity of aims was reported. The main aim was the diagnosis [3,17,19,20,21,22,24,26,27,28,29,32,34,35,36,38,39,40,41,44,45,46,48,52,53,54]. The second aim was to use this examination to monitor and to have the outcome of a particular treatment for TLF [23,25,30,33,42,49,50,51]. The third aim was the evaluation of movement as feedback during a particular task or for the evaluation of TLF changes in relation to movement [18,31,37,43,48,51,52].

### 3.9. Risk of Bias Assessment and Applicability Concern

**Figure 2:**
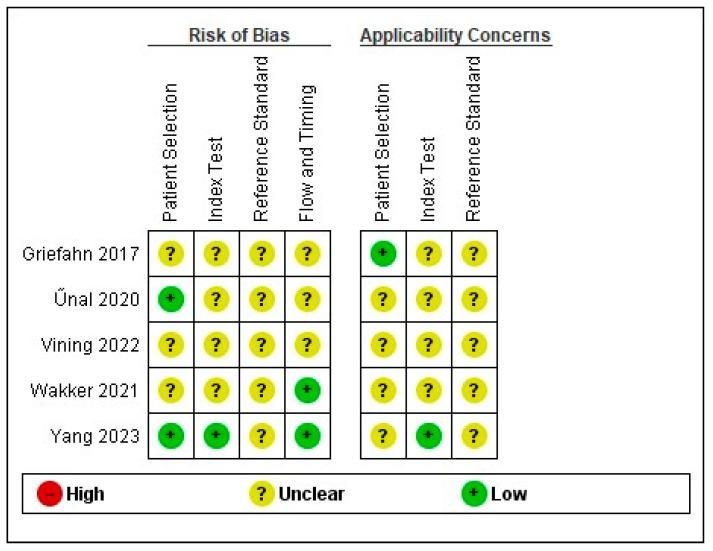
Risk of bias and applicability concerns summary about RCT studies: review authors’ judgements about each domain for each included study [25,30,38,42,50].

**Figure 3:**
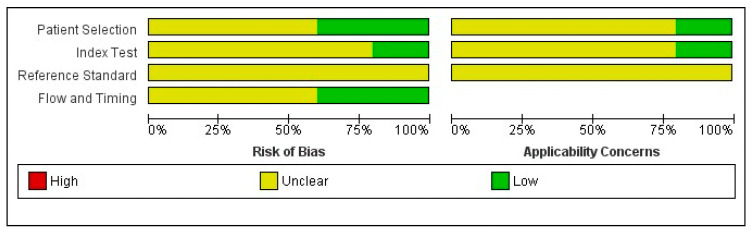
Risk of bias and applicability concerns graph about RCT studies: review authors’ judgements about each domain presented as percentages across included studies.

The total number of RCT studies (k = 100%) were judged to be unclear for reference standard. More than half of studies (k = 55%) were assessed with unclear risk of selection bias (Figure 2 and Figure 3). 

NOS scores of the included studies articles are shown in Table 4 and Table 5. After evaluation by two researchers, the studies received an average NOS score of 3.0, indicative of low-quality studies (Table 4 and Table 5). 

JBI Critical Appraisal Checklist for Case Reports studies was used to assess the quality of case reports (Table 6).

## 4. Discussion

To the author’s knowledge, this is the first and only systematic review selecting studies about the application of US imaging in TLF examination. The present systematic review is currently the largest collection of work from inception to April 2024 on the use of US imaging for TLF examination, and includes not only RCT, but also observational studies, case-control studies, case series, case reports and reviews. Given the methodological imperfections, unclear aspects, and heterogeneity of the investigations considered, this systematic review could not draw definitive conclusions, but certainly identifies some interesting clinically relevant points on a topic which has been exponential growth in recent years [1]. Thoracolumbar fascia role as tension-distributing structure means that any alterations in its mechanical properties can have significant repercussions [8,9,10,11]. For instance, the weakening of the multifidus muscle, a common phenomenon in patients with LBP, can lead to a reduction in the tensile strength of the TLF [8]. For this reason, the different studies included in this systematic review described various anatomical relationships involving the TLF and its correlations with surrounding structure. These included the fascial layers of TLF, the posterior layer specifically, the superficial layer of the deep fascia of the back, connections with lumbar multifidus, and erector spinae muscles, as well as the diaphragm, transversus abdominis, and internal oblique muscles [3,17,18,19,20,21,22,23,24,25,26,27,28,29,30,31,32,33,34,35,36,37,38,39,40,41,42,43,44,45,46,47,48,49,50,51,52,53]. Moreover, connections involving the fascia lata, plantar fascia, various components of the lumbar spine, and STF/SMF were assessed [3,17,18,19,20,21,22,23,24,25,26,27,28,29,30,31,32,33,34,35,36,37,38,39,40,41,42,43,44,45,46,47,48,49,50,51,52,53]. Furthermore, several terminologies were used to describe the deep fascial layers such as perimuscular connective tissue, paraspinal muscular compartment, etc.

Ultrasound technology, renowned for its affordability and portability, has revolutionized diagnostic imaging in daily practice [1]. The relatively low cost of ultrasound machines, compared to other imaging modalities such as MRI or CT scans, makes it a highly accessible tool for a wide range of healthcare settings [1]. In fact, also for the US examination of TLF, the authors of the included studies used a multiplicity of US devices and different types of transducers. Moreover, the TLF was assessed mainly with linear array transducers [3,17,18,20,21,22,23,24,25,26,27,28,29,30,34,35,36,38,39,40,41,43,44,45,47,48,49,50,51,52,53,54], but also with curvilinear array transducers [24,26,31,34,35,36,39,40,53]. Regarding the frequency and depth of acquisition, the majority of the articles examined offered frequency ranges for the ultrasound transducers, typically averaging from 4 MHz to 15 MHz for linear probes and from 2 MHz to 7 MHz for curvilinear probes. However, details of specific acquisition frequencies were seldom provided. B-mode was most commonly used across the majority of study, followed by B-mode with elastography to evaluate the TLF stiffness.

Ultrasound TLF assessment was predominately conducted with subjects in the prone position [17,18,19,20,21,23,27,30,32,38,39,41,42,43,44,45,46,49,51,52,53,54], with additional protocols incorporating various task or movements to assess dynamic changes. Protocols ranged from passive positioning to active tasks such as knee flexion or trunk extension. Probe handling was essential to the proper performance of an accurate and repeatable US examination. For example, Kellis et al. [18] conducted measurements during both rest (passive condition) and submaximal isometric knee flexion efforts (active condition), maintaining a neutral hip position while varying knee flexion angles at 0°, 45°, and 90°. Chen et al. [37] evaluated TLF across seven postures: rest, sitting, sitting-forward at 30° and 60°, standing, and standing-forward at 30° and 90°. Brandl et al. [19] used a digital goniometer to perform a controlled 60-degree thoracolumbar flexion followed by trunk extension over 8 s. Ultrasound assessment of the TLF was conducted at movement initiation and completion, akin to a deadlift. Dynamic US measurements of TLF displacement were performed between the junction of the latissimus dorsi muscle and a marked reference point using reflective tape [20]. Vininig et al. [21] positioned the probe parallel and 2–3 cm lateral to the L2–L3 spinous process interspace for optimal visibility of fascial tissue layers. Participants lay prone, moving their lower extremities downward by 15° for five cycles at 0.5 Hz. Pirri et al. [22] positioned the ultrasound transducer approximately 2–3 cm lateral to the L3 spinous process while the patient lay relaxed in the prone position, evaluating in transversal and longitudinal axis. Devantery [23] recommended bilaterally positioning the probe 2 cm lateral to L2–L3 interspace. Perez et al. [24] conducted ultrasound evaluation at the L4 vertebral level longitudinally over the anterior subcostal region with the patient supine on a 45° inclined bed. Chen et al. [40], Blain et al. [43], and Langevin et al. [48] also focused on L3–L4 lumbar vertebral levels. TLF was extended to the L4–L5 lumbar level by Bishop et al. [52] and Langevin et al. [54], while Panagos et al. [47] targeted the L5–S1 vertebral level. The most optimal point for TLF evaluation consistently emerged as L2–L3 [21,22,23,27,30,32,38,39,41,45,49,51,52,53,54]. In this context, potential dissimilarities in the protocols may potentially elucidate the difficulty in the comparison of the data.

Additionally, variations might arise due to the differences in the assessed parameters. They included TLF thickness, echogenicity, stiffness, architectural organization, deformation, shear strain, and displacement, providing comprehensive insights into TLF features. TLF thickness was the most evaluated parameter [3,17,18,19,22,23,24,25,31,32,33,34,39,40,44,46,48], followed by echogenicity [3,25,26,32,36]. Yerli et al. [17] reported a thickening of TLF in subjects with scoliosis, whereby, in the presence of chronic low back pain (LBP), it was intensified. Moreover, Pirri et al. [22] showed an increase of the TLF thickness in the longitudinal and transverse axes in chronic non-specific LBP patients. Vita et al. [44] reported that TLF was thicker in nonusers of hormonal contraceptives (*p =* 0.011), highlighting the influence of hormonal changes during the menstrual cycle on deep fasciae. Brandl et al. [20] showed TLF deformation in athletes and non-athletes with and without acute LBP, evaluating TLF deformation between latissimus dorsi muscle junction and an artificial reference. Different authors reported the TLF stiffness [18,23,28,37,38,41,43] in different spin positions. Chen et al. [37] used shear-wave elastography to study the relationship between shear modulus and different body postures of TLF, proving seven postures: rest, sitting, sitting-forward 30°, sitting forward 60°, standing, standing-forward 30°, and standing-forward 60°. Moreover, about TLF displacement, Langevin et al. [54] quantified shear plane motion within the TLF using ultrasound elasticity imaging in human subjects with and without CLBP.

Nonetheless, there remains a necessity for studies that examine the reproducibility across different systems and protocols to ascertain the reliability of TLS US measurements. In fact, only thirteen studies assessed the reliability of TLF Us measures, with intra-rater reliability consistently reported as good to excellent, and inter-rater reliability indicating excellent agreement. The possibility to compare the various data from different studies is tied to the need to use reliable protocols, as demonstrated by Yerli et al. [17], who used a published protocol [22], leading them to greater methodological rigor and enabling data comparison. Pirri et al. [22] reported that the intra-rater reliability for US TLF thickness assessement was for long axis TLF US examination (CNLBP: ICC_3,k_: 0.91; HV: ICC_3,k:_ 0.92) and for transverse axis:(CNLBP: ICC_3,k:_ 0.88; HV: ICC_3,k_: 0.88). Moreover, Yang et al. [25] showed an optimal inter-reliability ICC: 0.91. Finally, Wakker et al. [38] and Chen et al. [41] also reported an optimal inter-rater reliability in the assessment of TLF stiffness by shear-wave elastography, respectively ICC: 0.93 and ICC: 0.90–0.94.

Several limitations of the referenced studies should be noted. They are related to the study design and the lack of high-quality trials, as 100% of the identified RCT studies were judged to be at unclear for reference standard and more than half of studies (k = 55%) were assessed with unclear risk of selection bias. As mentioned above, most of them were carried out with different protocols. However, terminologies gap between studies seems to be a relevant limitation and makes translation of result difficult. This extreme heterogeneity leads to difficulty in carrying out a quantitative analysis and negatively affect the level of evidence. Finally, in the future, more high-quality clinical trials should be developed to ensure the viability of the proposed parameters, and to apply the assessment in the daily practice, standardizing these important technical issues.

## 5. Conclusions

In the past decade, there has been a notable increase in research focused on US imaging of TLF. The comprehensive analysis sheds light on the diverse applications and methodologies employes in TLF US research. This systematic review elucidates the utility of advanced ultrasound techniques in investigating TLF characteristics and functions. Studies aimed primarily at TLF diagnosis, treatment monitoring, or evaluating movement-related changes, underscoring the diverse clinical applications. Advanced US imaging holds promise as a reliable tool in musculoskeletal assessment, offering insights into TLF pathology/disfunction, treatment outcomes, and movement dynamics.

## Figures and Tables

**Table 1:** Description of the PICO (P= Population, I = Intervention, C = Comparison, O = Outcome) elements.

Population	Patients or healthy volunteers who underwent Ultrasound Imaging of Thoracolumbar fascia
Intervention	Ultrasound Imaging
Comparison	Ultrasound Imaging of other types of fasciae
Outcome	Parameters of thickness, echogenicity, stiffness, displacement

**Table 4:** Study of quality assessment using Newcastle-Ottawa scale for observational studies. Each asterisk represents if individual criterion within the subsction was fullfilled.

References	Selection				Comparability (Matched Analysis)	Assessment of Outcome	Outcomes	Adequacy of Follow-Up of Cohorts	NOS Score
	Consecutive or Obviously Representative Series of Cases	Representativeness of Exposed Cohort	Ascertainment of Exposure	Demonstration That Outcome of Interest Was Not Present at the Start of Study			Follow up Long Enough for the Outcome		
Yerli [17]	*	*	*	-	**	*	-	-	6
Kellis [18]	-	*	*	-	-	*	-	-	3
Gumruk [19]	*	*	*	*	**	*	-	-	6
Vining [21]	*	*	*	*	-	*	-	-	5
Pirri [22]	*	*	*	*	**	*	-	-	7
Devantery [23]	-	-	*	*	-	*	-	-	3
Perez [24]	*	*	*	-	*	*	-	-	5
Larivière [26]	-	*	-	-	-	*	-	-	2
Tamartash [27]	*	*	*	*	*	*	-	-	6
Bartsch [28]	-	-	-	-	-	*	-	-	1
Turan [31]	-	-	*	-	-	*	-	-	2
Ushida [32]	-	-	*	-	-	*	-	-	2
Larivière [34]	-	*	-	-	*	*	-	-	3
Larivière [36]	-	*	-	-	*	*	-	-	3
Chen [37]	-	-	*	-	*	*	-	-	3
Larivière [40]	-	*	*	-	*	*	-	-	4
Chen [41]	-	-	-	-	*	*	-	-	2
Blain [43]	-	-	-	-	*	*	-	-	2
Vita [44]	-	-	*	-	*	*	-	-	3
De Coninck [45]	-	*	*	-	*	*	-	-	4
Langevin [48]	-	-	*	-	*	*	-	-	3
Wong [49]	-	-	-	-	*	*	-	-	2
Tu [51]	-	-	-	-	*	*	-	-	2
Bishop [52]	-	-	*	-	-	*	-	-	2
Langevin [54]	-	*	*	-	*	*	-	-	4

**Table 5:** Study of quality assessment using Newcastle-Ottawa scale for case-control studies. Each asterisk represents if individual criterion within the subsction was fullfilled.

References	Selection				Comparability of Cohorts	Ascertainment of Exposure	Outcomes	Non-Response Rate	NOS Score
	Adequate Case Definition	Representativeness of Cases	Selection of Controls	Definition of Controls			Same Method of Ascertainment		
Brandl [20]	*	*	*	*	*	*	*	-	7
Brandl [29]	*	-	*	-	-	*	-	-	3
Venkatesan [33]	*	-	*	*	*	*	-	-	5

**Table 6:** Study of quality assessment using JBI Critical Appraisal Checklist for Case Reports studies.

References	Were Patient’s Demographic Characteristics Clearly Described?	Was the Patient’s History Clearly Described and Presented as a Timeline?	Was the Current Clinical Condition of the Patient on Presentation Clearly Described?	Were Diagnostic Tests or Assessment Methods and the Results Clearly Described?	Was the Intervention(s) or Treatment Procedure(s) Clearly Described?	Was the Post-Intervention Clinical Condition Clearly Described?	Were Adverse Events (Harms) or Unanticipated Events Identified and Described?	Does the Case Report Provide Takeaway Lessons?
Pirri [35]	Y	Y	Y	Y	Y	Y	-	Y
Fullerton [46]	Y	N	Y	Y	-	-	-	-
Panagos [47]	Y	N	Y	Y	-	-	-	-

## Data Availability

The data presented in this study are available upon request from the corresponding authors. The data are not publicly available due to privacy.
